# Effect of Weather on COVID-19 Transmission and Mortality in Lagos, Nigeria

**DOI:** 10.1155/2020/2562641

**Published:** 2020-08-18

**Authors:** Christian Ogaugwu, Hammed Mogaji, Euphemia Ogaugwu, Uchechukwu Nebo, Hilary Okoh, Stanley Agbo, Andrew Agbon

**Affiliations:** ^1^Department of Animal and Environmental Biology, Federal University Oye-Ekiti, Oye, Ekiti State, Nigeria; ^2^Department of Pharmacology, University of California, Irvine, CA, USA; ^3^Department of Microbiology, Federal University Oye-Ekiti, Oye, Ekiti State, Nigeria

## Abstract

The novel coronavirus disease 2019 (COVID-19) has become a global pandemic with more than 4 million confirmed cases and over 280,000 confirmed deaths worldwide. Evidence exists on the influence of temperature and humidity on the transmission of related infectious respiratory diseases, such as influenza and severe acute respiratory syndrome (SARS). This study therefore explored the effects of daily temperature and humidity on COVID-19 transmission and mortality in Lagos state, the epicenter of COVID-19 in Nigeria. Correlation analysis was performed using incidence data on COVID-19 and meteorological data for the corresponding periods from 9^th^ March to 12^th^ May, 2020. Our results showed that atmospheric temperature has a significant weak negative correlation with COVID-19 transmission in Lagos. Also, a significant weak negative correlation was found to exist between temperature and cumulative mortality. The strength of the relationship between temperature and the disease incidence increased when 1 week and 2 weeks' predetection delays were put into consideration. However, no significant association was found between atmospheric humidity and COVID-19 transmission or mortality in Lagos. This study contributes more knowledge on COVID-19 and will benefit efforts and decision-making geared towards its control.

## 1. Introduction

The novel coronavirus disease 2019 (COVID-19) is currently a global pandemic. This infectious respiratory disease was first reported in Wuhan, China, in December 2019 [1]. Symptoms commonly associated with COVID-19 include cough, fever, fatigue, and breathing difficulties, but sore throat, diarrhea, muscle pain, nasal congestion, and new loss of taste or smell may also occur [1, 2]. According to [3], there were 4,098,018 global confirmed cases and 283,271 confirmed deaths worldwide as of 12^th^ May 2020.

Many respiratory viral infectious diseases such as those caused by the human respiratory syncytial virus (RCV), influenza virus, and human coronaviruses show seasonal oscillation and are prevalent during winter [4]. Transmission of influenza was found to increase in colder and drier conditions [5]. In addition, the severe acute respiratory syndrome (SARS), caused by the coronavirus SARS-CoV, is affected by temperature [6]. The fact that SARS and COVID-19 are caused by coronaviruses and the outbreak of both diseases started during the winter seem to suggest that winter conditions could be promoting transmission of these infections [4, 7, 8].

Several groups have been investigating how weather components like temperature and humidity can influence the transmission and mortality from COVID-19. A study in China that looked at about 429 cities revealed that temperature can change COVID-19 spread [9], while another study in Jakarta, Indonesia, found that temperature was correlated with COVID-19 transmission, whereas humidity did not have any correlation [10]. However, another study by Yao et al. [11] in 224 Chinese cities found no association between temperature or relative humidity and COVID-19 spread. Ma et al. [12] also investigated COVID-19 mortality and weather in Wuhan, China, and observed that temperature had a positive association with deaths, whereas relative humidity had a negative association with COVID-19 deaths. COVID-19 is still recent and there is limited knowledge on it. More studies are still needed to better understand this novel disease.

Nigeria witnessed its first case of COVID-19 on the 27^th^ of February 2020. There have been 4,787 confirmed cases and 158 confirmed deaths as of 12^th^ May, 2020 [13]. In this study, we explored how daily atmospheric temperature and humidity affect COVID-19 transmission in Lagos state, the epicenter of the disease. This study contributes to the existing knowledge on this new disease and will aid efforts and decision-making geared towards its control.

## 2. Methods

### 2.1. Study Locations

Lagos state is the economic capital of Nigeria. It is located in south western Nigeria and lies within latitude 6°35′N and longitude 3°45′E [14]. The state is bordered to the south by the Atlantic Ocean and has the busiest sea port in Nigeria. To the west, Lagos state is bordered by the Republic of Benin and the Nigeria-Benin border is arguably the busiest border in the country as it is the gateway to other West African countries. Lagos also has the busiest international airport in Nigeria. Consequently, there is very high air, land, and sea traffic of people and goods in Lagos state. The state covers an area of about 3,577 km^2^ [14] and has a population estimated to be above 30 million people [15].

### 2.2. Data Collection

Incidence data on COVID-19 from 9^th^ March to 12^th^ May 2020 was obtained from the Lagos state Ministry of Health (https://twitter.com/lsmoh?lang=en). Meteorological data such as atmospheric temperature and humidity for the periods corresponding to COVID-19 incidences were obtained from the weather section of Time and Date AS (https://www.timeanddate.com/weather/).

### 2.3. Data Analysis

Data obtained were imported into Microsoft Excel 2017 for trendline analysis. Thereafter, the data were also imported into SPSS 20.0 statistical software for descriptive and inferential statistics. The relationship between meteorological variables (temperature and humidity) and COVID-19 incidence/transmission or mortality was analyzed using the Spearman correlation test with the formula:

(1) $$\begin{array}{c} r_{s}=\frac{\mathrm{cov}\left(rg_{x}\times rg_{y}\right)}{\sigma_{rgx}\sigma_{rgy}}, \end{array}$$

where *r*_*s*_ is the Spearman correlation, cov(*rg*_*x*_ × *rg*_*y*_) is the covariance of the rank variables, and *σ*_*rgx*_*σ*_*rgy*_ are the standard deviations of the rank variables.

Significance level was set at 95% probability values (*p* < 0.05).

## 3. Results and Discussion

The first case of COVID-19 in Nigeria was confirmed in Lagos on 27^th^ February 2020. While this index case was imported, the first case of local transmission (a contact of the index case) was confirmed on the 9^th^ of March and the state has since remained the epicenter of the disease with a total of 2006 confirmed cases as of 12^th^ May 2020 when data collection for this study was stopped.

**Figure 1:**
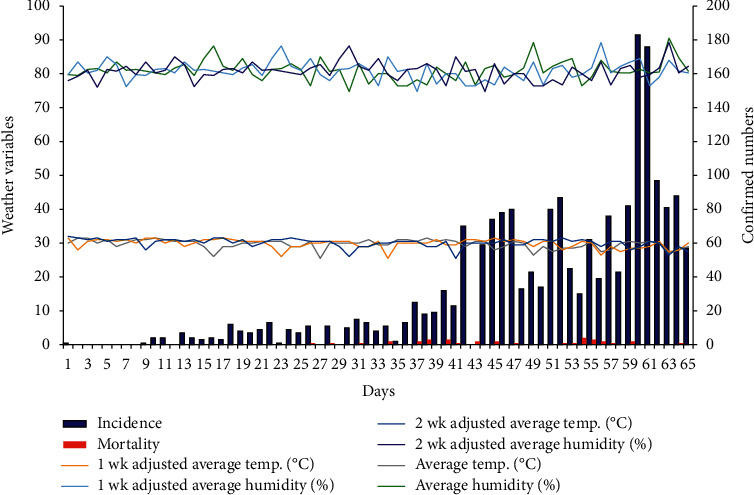
Temporal pattern of COVID-19 incidence, mortality, and meteorological factors (temperature and humidity) between 9^th^ March and 12^th^ May 2020 in Lagos state.

During the period of this study (9^th^ March to 12^th^ May 2020), Lagos state recorded a daily maximum incidence of 183 COVID-19 confirmed cases (Table 1; Figure 1). There were days when no single case was confirmed. A steady increase in the number of daily confirmed cases could be observed several days after the initial confirmed cases (Figure 1), implying that some community transmission had started to take place. The average minimum and maximum atmospheric temperatures in Lagos within this period were 26.00 ± 1.66°C and 33.38 ± 1.43°C, whereas the average minimum and maximum humidity were 67.42 ± 4.90% and 91.14 ± 2.80% (Table 1). There was a cumulative incidence of 2006 COVID-19 confirmed cases and a cumulative mortality of 34 COVID-19-related deaths.

Table 2 shows the result of the correlation test between COVID-19 transmission and mortality in Lagos state and the meteorological factors, temperature and humidity. A significant weak negative correlation exists between atmospheric temperature (maximum and average temperature) and daily incidence of COVID-19 (*r* = −0.356 and −0.327; *p* < 0.05). The same significant weak negative correlation existed between temperature (minimum, maximum, and average temperature) and cumulative incidence of COVID-19 (*r* = −0.302, −0.359, and −0.416; *p* < 0.05). There was no significant correlation between temperature and daily mortality. However, there was a significant weak negative correlation between temperature (minimum, maximum, and average temperature) and cumulative mortality (*r* = −0.255, −0.305, and −0.316; *p* < 0.05). No significant relationship was found to exist between humidity (minimum, maximum, and average) and COVID-19 transmission or mortality in Lagos.

COVID-19 symptoms typically start within 1–14 days after exposure to an infected person [1], and laboratory confirmation of positive cases could take additional days too. As such, the temperature and humidity on the day of positive confirmation of a case are not ideal parameters for correlational analysis. Considering this caveat, predetection date adjustments of 1 week and 2 weeks, respectively, were made for temperature and humidity, and the adjusted values were used to determine if there was any relationship with disease transmission. The correlation tests show that there was still a significant weak correlation between 1- and 2-week adjusted average temperature and daily COVID-19 transmission in Lagos (*r* = −0.357, −0.384; *p* < 0.05), as well as for cumulative incidence (*r* = −0.416, −0.460; *p* < 0.05) (Table 2). It is interesting to note that the weak negative correlation observed gets stronger within the 1 week to 2 weeks' predetection delay period, consistent with the disease incubation window period mentioned above. Furthermore, there was no significant correlation between both adjusted average temperatures and daily COVID-19 mortality, but a significant weak negative correlation existed between both adjusted average temperature and cumulative mortality (*r* = −0.373, −0.453; *p* < 0.05) (Table 2). No significant correlation whatsoever occurred between any of the adjusted humidity values and COVID-19 transmission or mortality in Lagos.

The findings of this study are consistent with those from Shi et al. [16] who also found a negative correlation between temperature and COVID-19 transmission in China, while at the same finding no correlation between humidity and the disease. Also, another study in Jakarta, Indonesia, reported an association between COVID-19 spread and temperature, but no association between the disease and humidity [10]. While this study and the two aforementioned studies were on a relatively microlevel, they concur with the findings of a macrolevel investigation by Demongeot and colleagues [17]. The large-scale study examined data from 21 different countries including France, Italy, Germany, Iran, Spain, Malaysia, Australia, the United Kingdom, and the United States of America and found out that temperature decreased the early spread of the disease [17]. It remains to be seen how seasonal temperatures will affect COVID-19.

The results from this study also differed in some aspects with other studies. Qi et al. [18] found a negative correlation between average daily temperature and COVID-19 cases in mainland China similar to this study, but they observed a correlation between humidity and the disease as well. Yao et al. [11] investigated COVID-19 in about 224 cities in China and failed to find any correlation between the disease incidence and temperature or humidity. Correlation was found between COVID-19 mortality and temperature in Wuhan, as well as for mortality and humidity [12]. It could be that the different conditions and limitation at our study location and the study locations of these other studies may have affected the outcomes of analyses.

The following limitations may have influenced results from this study. First, the temperature range during the period of study was between 20°C and 35°C. Freezing temperatures or temperatures above 35°C may have some influence on COVID-19 transmission or mortality that our study would not be able to detect. Second, Lagos is a very highly populated state and the close contact among people could positively influence disease transmission. Third, incidence or mortality figures may be underreported due to social stigmatization and other factors that could lead to infected persons avoiding laboratory testing or sick persons going to hospitals or isolation centers. Fourth, meteorological data were accessed remotely from satellites or obtained from meteorological agencies or weather websites, but these data may contain varying inaccuracies due to human or machine errors. Fifth, some COVID-19-related deaths also had underlying illnesses and the actual cause of death sometimes becomes unclear. Sixth, the novel coronavirus, SARS-CoV-2, that causes COVID-19 could be genetically evolving in different geographic locations due to mutations and might not be exactly the same. The recent sequencing of SARS-CoV-2 from the Nigerian index case revealed genomic clusters that belong to different geographic clades [19]. Future studies could determine if COVID-19 in different locations are due to the same strain of SARS-CoV-2 or if genomic mutations have somehow influenced their transmission and virulence. Lastly, COVID-19 is new and our study was conducted for a duration of about 2 months. Investigations lasting up to one year could reveal more about the disease and show if it exhibits seasonality like other respiratory diseases.

Deductions from this study include some of the followings: (i) Considering the weak correlation between COVID-19 spread and atmospheric temperature between 26.00 ± 1.66°C and 33.38 ± 1.43°C, conditions of higher temperatures (and possibly at equally high humidity) may have more profound negative effect on the disease transmission although such conditions might prove distressful or discomforting. (ii) COVID-19 may likely be nonseasonal in places with weather conditions similar to the ones under which this study was carried out. Efforts should therefore focus on finding suitable medications for treatment of COVID-19. While present efforts continue and progresses are made as some compounds have shown promise *in vitro* against SARS-CoV-2 [20], more research and trials are still needed. Adoption of effective preventive measures such as wearing masks and practicing social distancing will be important in communities to curtail the spread of the disease.

## 4. Conclusion

COVID-19 is new and more studies and time will be required to better understand it. This study found out that there is a weak negative relationship between temperature and the spread of this disease, as well as its related mortality. Higher temperatures might reduce the disease transmission. The weak relationship between atmospheric temperature and COVID-19 incidence and mortality suggests that the disease may exhibit little or no seasonality unlike SARS or influenza, at least in places with similar weather conditions as in this study.

## Figures and Tables

**Table 1:** Summary of COVID-19 pandemic and meteorological factors in Lagos, Nigeria (9^th^ March to 12^th^ May 2020).

Variables	Daily measures					
	Mean ± SD	Min	P_25_	P_50_	P_75_	Max
Incidence (new cases)	30.85 ± 39.79	0.00	3.00	12.00	51.00	183.00
Cumulative incidence	453.82 ± 571.42	2.00	30.50	166.00	763.00	2006
Mortality	0.52 ± 0.95	0.00	0.00	0.00	1.00	4.00
Cumulative mortality	10.49 ± 12.23	0.00	0.00	3.00	19.00	34.00
Minimum temperature (°C)	26.00 ± 1.66	20.00	25.00	26.00	27.00	28.00
Maximum temperature (°C)	33.38 ± 1.43	29.00	33.00	34.00	34.00	35.00
Minimum humidity (%)	67.42 ± 4.90	59.00	64.00	66.00	70.50	83.00
Maximum humidity (%)	91.14 ± 2.80	85.00	89.00	91.00	93.00	97.00
Average temperature (°C)	29.71 ± 1.33	25.50	29.00	30.00	30.50	31.50
Average humidity (%)	81.04 ± 2.92	74.75	79.75	80.75	82.38	90.50
1 wk adjusted average temperature (°C)	29.84 ± 1.33	25.5	29.00	30.00	30.75	31.50
1 wk adjusted average humidity (%)	80.89 ± 2.77	74.75	79.63	81.00	82.63	89.25
2 wk adjusted average temperature (°C)	30.09 ± 1.31	25.50	29.50	30.50	31.00	32.00
2 wk adjusted average humidity (%)	80.73 ± 2.72	74.75	79.50	81.00	82.25	89.25

**Table 2:** Spearman's correlation test between meteorological factors and COVID-19 cases in Lagos, Nigeria.

	New cases	Cum. cases	Mort.	Cum. mort.	Min. temp. (°C)	Max. temp. (°C)	Avg. temp. (°C)	1 wk. adj. avg. temp (°C)	2 wk. adj. avg. temp. (°C)	Min hum. (%)	Max. hum. (%)	Avg. hum. (%)	1 wk. adj. avg. hum. (%)	2 wk. adj. avg. hum. (%)
New cases	1													
Cum. cases	0.887^*∗*^ (0.000)	1												

Mortality	0.361^*∗*^ (0.003)	0.420^*∗*^ (0.000)	1											

Cum. mortality	0.853^*∗*^ (0.000)	0.971^*∗*^ (0.000)	0.450^*∗*^ (0.000)	1										

Min. temp. (°C)	−0.217 (0.082)	−0.302^*∗*^ (0.015)	−0.038 (0.764)	−0.255^*∗*^ (0.040)	1									

Max. temp. (°C)	−0.356^*∗*^ (0.004)	−0.359^*∗*^ (0.003)	−0.049 (0.699)	−0.305^*∗*^ (0.013)	0.485^*∗*^ (0.000)	1								

Avg. temp. (°C)	−0.327^*∗*^ (0.008)	−0.372^*∗*^ (0.002)	−0.030 (0.811)	−0.316^*∗*^ (0.010)	0.859^*∗*^ (0.000)	0.843^*∗*^ (0.000)	1							

1 wk. adj. avg. temp. (°C)	−0.357^*∗*^ (0.003)	−0.416^*∗*^ (0.001)	−0.240 (0.054)	−0.373^*∗*^ (0.002)	0.029 (0.818)	0.143 (0.257)	0.057 (0.649)	1						

2 wk. adj. avg. temp. (°C)	−0.384^*∗*^ (0.002)	−0.460^*∗*^ (0.000)	−0.232 (0.062)	−0.453^*∗*^ (0.000)	−0.065 (0.604)	−0.042 (0.741)	−0.052 (0.681)	0.81 (0.519)	1					

Min hum. (%)	0.157 (0.213)	0.147 (0.244)	−0.011 (0.933)	0.146 (0.247)	−0.241 (0.053)	−0.732^*∗*^ (0.000)	−0.577^*∗*^ (0.000)	−0.052 (0.680)	0.095 (0.449)	1				

Max. hum. (%)	0.142 (0.258)	0.077 (0.540)	−0.116 (0.356)	0.045 (0.720)	−0.526^*∗*^ (0.000)	−0.244 (0.050)	−0.458^*∗*^ (0.000)	−0.143 (0.257)	0.124 (0.324)	0.103 (0.412)	1			

Avg. hum. (%)	0.060 (0.637)	0.019 (0.881)	−0.211 (0.091)	−0.006 (0.962)	−0.378^*∗*^ (0.002)	−0.644^*∗*^ (0.000)	−0.597^*∗*^ (0.000)	−0.051 (0.687)	0.111 (0.377)	0.731^*∗*^ (0.000)	0.509^*∗*^ (0.000)	1		

1 wk. adj. avg. hum. (%)	−0.014 (0.912)	−0.062 (0.625)	−0.001 (0.995)	−0.092 (0.465)	0.043 (0.735)	−0.041 (0.747)	0.022 (0.861)	−0.527^*∗*^ (0.000)	0.026 (0.838)	0.011 (0.931)	0.209 (0.094)	0.083 (0.510)	1	

2 wk. adj. avg. hum. (%)	−0.126 (0.318)	−0.023 (0.858)	0.050 (0.691)	−0.040 (0.753)	0.070 (0.581)	0.024 (0.852)	0.035 (0.779)	0.041 (0.748)	−0.531^*∗*^ (0.000)	−0.087 (0.492)	−0.010 (0.935)	0.005 (0.971)	0.022 (0.861)	1

Cum. cases, cumulative cases; cum. mort., cumulative mortality; min. temp., minimum temperature; max. temp., maximum temperature; avg. temp., average temperature; 1 wk. adj. avg. temp., 1-week adjusted average temperature; 2 wk adj. avg. temp., 2-week adjusted average temperature; min. hum., minimum humidity; max. hum., maximum humidity; avg. hm., average humidity; 1 wk. adj. avg. hum., 1-week adjusted average humidity; 2 wk. adj. avg. hum., 2-week adjusted average humidity. Correlation values with asterisk show significant correlation at a probability level of 95%, *p* < 0.05. *p* values are in brackets.

## Data Availability

Data used for this study can be accessed as described in Section 2.2 (data collection) from the Lagos state Ministry of Health (https://twitter.com/lsmoh?lang=en) and from Time and Date AS (https://www.timeanddate.com/weather/). Harmonised data from these sources are available online as Supplementary Materials.

## Supplementary Materials

Supplementary Materials COVID-19 incidence and mortality in Lagos, Nigeria, and associated weather variables.
